# Preoperative Tapentadol Enhances the Depth of Anesthesia-Induced Sleep, Recovery Profile, and Serotonergic Modulation in Dogs Undergoing Ovariectomy with Propofol–Sevoflurane Anesthesia

**DOI:** 10.3390/vetsci13040378

**Published:** 2026-04-14

**Authors:** Giovanna Lucrezia Costa, Fabio Bruno, Fabio Leonardi, Nicola Maria Iannelli, Giuseppe Bruschetta, Suzane Lilian Beier

**Affiliations:** 1 Department of Veterinary Sciences, University of Messina, 98168 Messina, Italy; nicolamaria.iannelli@unime.it (N.M.I.); giuseppe.bruschetta@unime.it (G.B.); 2 Department of Veterinary Science, University of Parma, Via del Taglio 10, 43126 Parma, Italy; fabio.leonardi@unipr.it; 3 Surgery Department, Veterinary School, Minas Gerais State Federal University (UFMG), Av. Antônio Carlos 6627, Belo Horizonte 30123-970, Brazil; suzanelb@ufmg.br

**Keywords:** balanced analgesia, perioperative nociception control, anesthetic depth assessment, emergence quality, neurotransmitter regulation, small animal surgery

## Abstract

Background: Optimizing pain control and anesthesia stability is essential for surgical outcomes in dogs. This study evaluated the effects of preoperative tapentadol on anesthesia depth, recovery, and serotonin levels in dogs undergoing elective spaying. Methods: Sixty-six healthy female dogs were randomly assigned to three groups: standard pain relief, tapentadol alone, or tapentadol combined with standard pain relief. Anesthesia was induced with propofol and maintained with sevoflurane. Intraoperative heart rate, blood pressure, anesthetic depth, postoperative sedation, pain scores, and plasma serotonin concentrations were recorded. Results: Tapentadol-treated dogs showed deeper and more stable anesthesia during surgical stimulation, higher early postoperative sedation that resolved within two hours, and reduced postoperative serotonin levels compared with controls. All analgesic protocols maintained adequate pain control, and biochemical parameters remained within normal limits. Conclusions: Preoperative tapentadol enhances anesthetic stability, supports smoother early recovery, and modulates serotonin levels in dogs, suggesting benefits for perioperative pain management and overall surgical welfare.

## Simple Summary

Managing pain during and after surgery is very important for dogs’ health and comfort. This study looked at whether giving the pain-relief drug tapentadol before spaying surgery could improve how dogs respond to anesthesia and recover afterward. Dogs were given tapentadol, a standard pain reliever, or a combination of both. During surgery, researchers measured heart rate, blood pressure, and the depth of anesthesia. After surgery, they observed how sleepy or calm the dogs were and measured serotonin, a brain chemical linked to stress and sleep. Dogs that received tapentadol had deeper and more stable anesthesia, recovered more calmly in the first hours after surgery, and showed changes in serotonin that may indicate lower stress. The study shows that giving tapentadol before surgery can make the procedure safer and more comfortable, support better recovery, and help veterinarians provide effective pain management. These findings can improve the overall welfare of dogs undergoing routine surgical procedures.

## 1. Introduction

Optimizing perioperative analgesia remains a central objective of modern veterinary anesthesiology, as it directly influences anesthetic stability, postoperative recovery, and the overall welfare of surgical patients [1,2]. In canine practice, elective ovariectomy represents a widely used and standardized surgical model for evaluating perioperative nociception and anesthetic management. Surgical trauma activates a complex neuroendocrine stress response involving inflammatory mediators, autonomic activation, and neurochemical changes within central nociceptive pathways. If inadequately controlled, this cascade may promote central sensitization and contribute to prolonged postoperative discomfort or chronic postsurgical pain [3]. Beyond nociceptive control, increasing attention has been directed toward the interaction between anesthesia, pain, and sleep physiology. Sleep is a fundamental biological process involved in tissue repair, immune regulation, metabolic homeostasis, and neuronal plasticity [4]. Surgical stress and nociceptive signaling can disrupt normal sleep architecture, producing fragmented sleep, sympathetic activation, and impaired recovery. Conversely, sleep deprivation lowers nociceptive thresholds and amplifies inflammatory responses, creating a bidirectional interaction between pain and sleep disturbance [5,6]. General anesthesia induces a reversible state of unconsciousness often described as a “sleep-like” condition; however, this state differs from physiological sleep in several neurophysiological aspects. Anesthetic agents modify thalamocortical connectivity and produce characteristic electroencephalographic (EEG) patterns that partially resemble slow-wave sleep but lack the normal cyclicity and homeostatic regulation of natural sleep [7,8]. In particular, GABAergic and volatile anesthetics such as propofol and sevoflurane generate high-amplitude slow-delta oscillations comparable to deep non-rapid eye movement (NREM) sleep. These oscillations are considered markers of anesthesia-induced sleep depth, reflecting the degree of cortical synchronization and hypnotic stability achieved during anesthesia [9].

The depth and continuity of anesthesia-induced sleep have recently emerged as important determinants of perioperative outcomes. Stable slow-wave activity during anesthesia is associated with improved autonomic balance, reduced nociceptive responsiveness, and more predictable emergence from anesthesia. Conversely, inadequate hypnotic depth may lead to intraoperative arousal responses, increased anesthetic requirements, and altered postoperative recovery patterns [10,11,12]. Consequently, pharmacological strategies capable of stabilizing neural oscillatory activity during anesthesia may enhance the quality of the anesthesia-induced sleep state and improve recovery trajectories. Monoaminergic neurotransmitters play a crucial role in regulating both physiological sleep and anesthetic-induced unconsciousness. Among these, serotonin (5-hydroxytryptamine, 5-HT) is a key neuromodulator involved in sleep–wake regulation, autonomic stability, nociceptive modulation, and neuroendocrine responses [13,14]. Serotonergic neurons located in the raphe nuclei project widely throughout the central nervous system and interact with thalamocortical circuits responsible for sleep synchronization. Experimental evidence suggests that serotonergic signaling contributes to the modulation of slow-wave activity and influences the transition between arousal and hypnotic states during anesthesia [15]. Changes in systemic serotonin levels may therefore reflect neurochemical adaptations occurring during anesthetic exposure. Monitoring perioperative serotonin dynamics could provide insight into central nervous system stability and the capacity of analgesic interventions to preserve neurochemical substrates involved in sleep regulation and autonomic homeostasis [16]. Tapentadol is a centrally acting analgesic characterized by a dual mechanism combining μ-opioid receptor agonism with norepinephrine reuptake inhibition (MOR–NRI). This pharmacological profile provides effective analgesia while reducing several adverse effects commonly associated with conventional opioids, including excessive sedation, gastrointestinal dysfunction, and respiratory depression [17,18]. The noradrenergic component enhances descending inhibitory pain pathways and contributes to autonomic stabilization, which may favor a more stable anesthetic state and improved hypnotic depth during anesthesia [19]. In veterinary medicine, pharmacokinetic studies have demonstrated that tapentadol is well absorbed in dogs and presents a half-life compatible with perioperative analgesic protocols, supporting its potential role within multimodal analgesic strategies [20]. However, its possible influence on the neurophysiological quality of anesthesia-induced sleep and on perioperative serotonergic modulation remains poorly investigated. Therefore, the primary aim of the present study was to evaluate the effect of preoperative tapentadol administration on the depth and quality of anesthesia-induced sleep, postoperative recovery profile, and systemic serotonin modulation in dogs undergoing elective ovariectomy under propofol–sevoflurane anesthesia. We hypothesized that preoperative tapentadol would enhance the stability and depth of anesthesia-induced sleep, support serotonergic neurochemical homeostasis, and promote improved postoperative recovery.

## 2. Materials and Methods

### 2.1. Ethical Approval and Study Design

This prospective, randomized clinical trial was approved by the Animal Welfare Ethics Committee of the University of Parma (approval code: 03/CESA/2023) and conducted in accordance with Regulation (EU) No. 536/2014, the European Directive 86/609/EEC (O.J. L 358/1, 18 December 1986), and U.S. Animal Welfare Assurance (No. A5594-01, Department of Health and Human Services, Washington, DC, USA). Written informed consent was obtained from all dog owners prior to enrollment. All study materials, protocols, and datasets generated during the current study are available from the corresponding author upon reasonable request. No restrictions apply to the availability of materials or data. An a priori sample size calculation was performed using G*Power (v3.1). The sample size calculation was based on an expected effect size (f = 0.45), α = 0.05, and power (1 − β) = 0.80 for a one-way ANOVA comparing three groups. Resulting in a required sample size of 66 dogs. Dogs were randomly allocated to the experimental groups using a computer-generated randomization sequence to ensure equal distribution among treatments.

### 2.2. Animals and Inclusion Criteria

Sixty-six clinically healthy female dogs scheduled for elective ovariectomy were enrolled. Dogs were classified as American Society of Anesthesiologists (ASA) physical status I based on physical examination, complete blood count, and serum biochemistry. Dogs not meeting ASA I criteria were excluded. All animals were housed under similar environmental conditions and allowed a short acclimation period before clinical procedures to minimize stress-related physiological variability. No specific breed exclusions were applied; however, brachycephalic breeds were not represented in the study population. Dogs were client-owned and maintained under their usual home conditions; a 30-min acclimation period was provided prior to clinical procedures.

### 2.3. Randomization and Experimental Groups

Group allocation was based on the specific preanesthetic analgesic protocol administered before induction of general anesthesia.

Group C: received meloxicam 0.2 mg kg^−1^ IM (Metacam^®^ Injectable Solution, Boehringer Ingelheim Animal Health, Milan, Italy) and atropine sulfate 0.03 mg kg^−1^ (Atropine Sulfate^®^, A.T.I. S.r.l., Bologna, Italy) IM as preanesthetic medication, administered 10 min before induction of general anesthesia.

Group TM: received oral tapentadol (Palexia^®^, tablets 50 mg, Grünenthal GmbH, Aachen, Germany) administered at 10 mg kg^−1^ 2 h before anesthesia, combined with meloxicam 0.1 mg kg^−1^ IM and atropine sulfate (1 mg mL^−1^ injectable solution) IM administered 10 min before induction as preanesthetic medication.

Group T: received oral tapentadol administered at 10 mg kg^−1^ 2 h before anesthesia and atropine sulfate (1 mg mL^−1^ injectable solution) IM administered 10 min before induction as preanesthetic medication.

### 2.4. Preoperative Management and Anesthesia

Food was withheld for 6 h and water for 2 h before anesthesia. Dogs were allowed a 30-min acclimation period before baseline physiological measurements were recorded. A 20 G × 32 mm catheter was placed in the cephalic vein for administration of lactated Ringer’s solution (5 mL kg^−1^ h^−1^) throughout anesthesia. Twenty minutes after premedication, anesthesia was induced with propofol (Proposure^®^ 1%, Merial, Milan, Italy) to effect. Propofol was administered intravenously in incremental boluses (approximately 0.5 mg/kg) until adequate conditions for intubation were achieved and dogs were endotracheally intubated with a cuffed tube. Anesthesia was maintained with sevoflurane (Sevoflo^®^, Zoetis, Catania, Italy) in 100% oxygen delivered via a circle rebreathing system. Sevoflurane concentration was adjusted as required to maintain an adequate anesthetic plane based on clinical parameters, rather than using predetermined vaporizer settings. End-tidal sevoflurane concentrations were continuously recorded in all dogs to monitor anesthetic depth and guide adjustments. Mechanical ventilation was provided using a pressure-controlled ventilator in synchronized intermittent mandatory ventilation (SIMV) mode (GE Datex Ohmeda Avance Ultramed, Italy) with the following settings:

Respiratory rate: 12 breaths min^−1^
Positive end-expiratory pressure (PEEP): 4 cm H_2_O
Inspiratory/expiratory ratio: 1:2
Peak airway pressure: 12 cm H_2_O

Physiological parameters, including heart rate, arterial blood pressure, respiratory rate, oxygen saturation, and body temperature, were continuously monitored throughout the procedure to ensure safety.

Postoperative analgesia consisted of meloxicam 0.1 mg kg^−1^ PO every 24 h in Groups C and TM. In Group T, meloxicam 0.2 mg kg^−1^ PO was administered 6 h after preoperative tapentadol, followed by meloxicam 0.1 mg kg^−1^ PO every 24 h thereafter.

### 2.5. Physiological and Anesthetic Monitoring

After the acclimation period, baseline values were recorded:

• Heart rate (HR)
• Respiratory rate (RR)
• Non-invasive arterial blood pressure (systolic, mean, diastolic)
• Rectal temperature

During anesthesia, end-tidal CO_2_ (EtCO_2_), End-tidal sevoflurane concentrations (ETSevo) peripheral oxygen saturation (SpO_2_), and inspired and expired anesthetic concentrations were continuously monitored using a multiparametric monitor (GE Datex Ohmeda Avance Ultramed, Padua, Italy).

Measurements were recorded at the following standardized time points: T0 (baseline), 20 min after premedication (P; except EtCO_2_ and anesthetic concentrations), post-induction (A), skin incision (SI), laparotomy (L), traction and exteriorization of the first and second ovary (TPI, TPII), and skin closure (SC). These time points were selected to correspond to the main nociceptive surgical stimuli occurring during elective ovariectomy.

### 2.6. Intraoperative Nociceptive Response Evaluation 

Intraoperative nociception was assessed using a cumulative pain score (CPS) based on percentage changes in heart rate (HR), respiratory rate (RR), and systolic arterial pressure relative to post-induction values. Respiratory rate was controlled by mechanical ventilation and remained constant; therefore, nociceptive assessment relied primarily on heart rate and blood pressure variations. Each variable was assigned a score from 0 to 4 according to the magnitude of variation: 0 = ≤0%; 1 = ≤10%; 2 = >10–20%; 3 = >20–30%; 4 = >30%. The CPS scoring system was adapted from previously published nociceptive assessment methods used in canine anesthetic studies. A cumulative pain score (CPS) ≥ 10, corresponding to an approximate 20% increase in the monitored physiological variables, was considered indicative of intraoperative nociception and triggered rescue analgesia with fentanyl (2 µg kg^−1^ IV) [21].

### 2.7. Assessment of Anesthetic Depth

Depth of anesthesia was clinically evaluated throughout the procedure using a composite scoring system based on palpebral reflex, corneal reflex, jaw tone, eye position, and pupil size. Each parameter was assigned a score ranging from 0 to 2, with increasing scores indicating a deeper plane of anesthesia. The individual scores were summed to obtain a total anesthetic depth score (range: 0–10). Higher scores corresponded to deeper planes of anesthesia and were used to standardize the clinical assessment of anesthetic depth during the experimental procedure [22].

Anesthetic depth scores were assigned by three independent observers who were blinded to treatment group allocation.

Clinical assessments were performed at the following predefined time points: at post-induction (A); skin incision (SI); laparotomy (L); traction and exteriorization of the first and second ovary (TPI, TPII); and skin closure (SC). The scoring criteria for each parameter were as follows:


**Parameter**

**Score 0**

**Score 1**

**Score 2**

**Palpebral reflex**
Brisk reflex presentReduced or sluggish reflexAbsent
**Corneal reflex**
PresentDecreasedAbsent
**Jaw tone**
Marked resistance to mouth openingModerate resistanceFlaccid, no resistance
**Eye position**
CentralVentromedial rotationCentral position associated with deep anesthesia
**Pupil size**
DilatedIntermediateConstricted

### 2.8. Postoperative Pain Assessment and Analgesia Evaluation

Postoperative pain was evaluated using the Colorado State University Canine Acute Pain Scale, (score 0–4), at extubation and every 6 h for 24 h. A pain score ≥ 2 triggered rescue analgesia with methadone (0.2 mg kg^−1^ IM). Postoperative analgesic management was scheduled according to the pharmacological treatments administered prior to anesthesia. This approach was designed to maintain effective analgesia while avoiding pharmacological overlap between preoperative and postoperative analgesic protocols.

### 2.9. Postoperative Sedation Assessment

Postoperative sedation was evaluated using a modified Richmond Agitation–Sedation Scale (RASS), adapted for use in dogs [23]. The scale allows standardized assessment of sedation depth based on spontaneous behavior and responses to verbal and tactile stimulation. Scoring was performed by an investigator blinded to the treatment allocation. Sedation scores ranged from 0 (alert and calm) to −5 (unarousable), with progressively more negative scores indicating deeper sedation. Behavioral descriptors adapted for canine patients were as follows:

• 0 Alert and calm: awake, responsive to environment and handling
• −1 Drowsy: reduced spontaneous activity but easily responsive to voice
• −2 Light sedation: briefly awakens to voice or gentle tactile stimulation
• −3 Moderate sedation: movement or eye opening only in response to repeated verbal or light physical stimulation
• −4 Deep sedation: minimal response to physical stimulation
• −5 Unarousable: no response to voice or physical stimulation

Sedation assessments were performed at predefined postoperative time points: immediately after extubation (R0), 30 min (R30), 60 min (R60), and 120 min (R120) after extubation, corresponding to the early recovery phase following general anesthesia induced with propofol and maintained with sevoflurane [23].

### 2.10. Hematological and Biochemical Analyses

Blood samples (5 mL) were collected from the cephalic vein at predefined time points. Samples were divided into serum tubes for biochemical analysis and K_3_-EDTA tubes for complete blood count. Serum was obtained by centrifugation at 1500 rpm for 15 min after 3 h at 4 °C. Biochemical analyses were performed at 37 °C using a UV–Vis spectrophotometer (A560, Fulltech, Rome, Italy). AST and ALT activities were measured kinetically, while serum glucose, albumin, total protein, and blood urea were quantified using spectrophotometry-compatible commercial kits: Glucose (GOD-PAP, Randox Laboratories Ltd., Crumlin, UK), Albumin (BCG, Gesan Production S.r.l., Trapani, Italy), Total Protein (Biuret, MTD Diagnostics S.r.l., Caserta, Italy), and Urea (urease–peroxidase/Berthelot, AMP Diagnostics, Graz, Austria). These biochemical parameters were evaluated to monitor potential systemic effects of anesthesia and perioperative pharmacological treatments.

### 2.11. Determination of Plasma 5-Hydroxytryptamine (5-HT)

For 5-HT analysis, 2 mL of blood was collected in K_3_-EDTA tubes and centrifuged within 2–3 h at 4500 rpm for 10 min at 4 °C to obtain platelet-poor plasma (PPP). PPP samples were stored at −20 °C until analysis. Blood samples for 5-HT determination were collected at baseline (T0), during anesthesia, and in the early postoperative period. Plasma 5-HT concentrations were measured using a commercially available ELISA kit (DEE5900, Demeditec Diagnostic GmbH, Kiel, Germany) kit according to the manufacturer’s instructions. Absorbance was read at 450 nm (reference 630 nm), and concentrations were calculated from a standard calibration curve and expressed as ng mL^−1^. All samples were analyzed in duplicate to ensure analytical reliability. Assay sensitivity was 0.5 ng mL^−1^, with intra- and inter-assay coefficients of variation of 6.2% and 9.1%, respectively.

### 2.12. Statistical Analysis

Statistical analyses were performed using IBM SPSS Statistics (version 27.1; IBM Corp., Milano, Italy). The normality of data distribution was assessed using the Shapiro–Wilk test. Continuous variables are presented as mean ± standard deviation (SD). Changes over time and differences among experimental groups were analyzed using repeated-measures analysis of variance (ANOVA), followed by Bonferroni post hoc multiple comparison tests when significant effects were detected. When required to satisfy model assumptions, logarithmic (log_10_) transformation of the data was automatically applied by the software. Clinical scores, including pain and sedation scores, were treated as ordinal variables and analyzed using the Kruskal–Wallis test followed by Dunn’s post hoc test for pairwise comparisons. Statistical significance was set at *p* < 0.05. Three independent observers performed all pain and sedation scoring. Kendall’s coefficient of concordance W was calculated for all scores to assess inter-observer agreement.

## 3. Results

### 3.1. Demographic and Baseline Characteristics

A total of 66 client-owned, clinically healthy female dogs were enrolled in the study and randomly allocated to three experimental groups (n = 22 per group). The mean age of the dogs was 2.0 ± 0.5 years, and the mean body weight was 18.0 ± 0.7 kg. All dogs were classified as American Society of Anesthesiologists (ASA) physical status I based on physical examination, complete blood count, and serum biochemistry. Baseline physiological parameters including heart rate (HR), respiratory rate (RR), non-invasive arterial blood pressure (systolic, mean, and diastolic), and rectal temperature were recorded after a 30-min acclimation period. No significant differences were observed between groups for any of these baseline variables (*p* > 0.05), no clinically relevant hypotension was observed in any group during anesthesia, and arterial blood pressure remained within physiological limits throughout the procedure confirming homogeneity of the study population prior to anesthesia. The randomization process distributed animals evenly among the three groups. All subjects were clinically healthy at enrollment, allowing comparability for subsequent intraoperative and postoperative assessments.

### 3.2. Intraoperative Nociceptive Response

All three experimental groups, Group C (Meloxicam + Atropine), Group TM (Tapentadol + Meloxicam + Atropine), and Group T (Tapentadol + Atropine), showed low CPS values throughout the procedure, with median total scores ranging from 1 to 2. No significant differences were observed between groups at any surgical time point (*p* > 0.05 for all comparisons).

No dogs required rescue analgesia (fentanyl 2 µg kg^−1^ IV) (Table 1).

### 3.3. Intraoperative Anesthetic Depth

Total anesthetic depth median scores, at post-induction were similar between groups (Group C; Group TM; Group T *p* = 0.12), indicating comparable depth of anesthesia immediately after induction.

During skin incision, laparotomy, and ovarian traction (TPI and TPII), dogs in the tapentadol-containing groups (TM and T) exhibited significantly higher anesthetic depth scores compared with Group C (*p* = 0.003 for SI and L; *p* = 0.001 for TPI and TPII).

At skin closure (SC), differences between groups were not statistically significant (*p* = 0.08), with median scores slightly higher in Groups TM and T compared to Group C.

Overall, median total anesthetic depth scores were higher in Groups TM and T compared with Group C (Table 2).

### 3.4. Postoperative Pain Assessment and Analgesia

Across all experimental groups, median pain scores did not exceed the rescue threshold (score ≥ 2) at any time point in any group, and no dogs required rescue analgesia with methadone (0.2 mg kg^−1^ IM).

Inter-observer agreement for pain scoring was high, with Kendall’s coefficient of concordance W = 0.91 (*p* < 0.001), confirming high reproducibility and reliability of the assessments performed by the three blinded observers (Table 3).

Pain was assessed using the Colorado State University Canine Acute Pain Scale by three trained observers blinded to group allocation. Rescue analgesia (methadone 0.2 mg kg^−1^ IM) was administered for scores ≥ 2; no dogs required rescue analgesia during the 24-h observation period. Values are expressed as median (range). Inter-observer agreement was excellent (Kendall’s W = 0.91, *p* < 0.001), confirming high reproducibility and reliability of the pain assessments.

### 3.5. Postoperative Sedation

Postoperative sedation at extubation (R0), dogs in the tapentadol-containing groups (TM and T) exhibited significantly deeper sedation compared with Group C (*p* = 0.004). This trend persisted at 30 min (R30), with Groups TM and T showing moderate sedation relative to Group C (*p* = 0.006).

By 60 min (R60), sedation scores had partially recovered but remained significantly lower in the tapentadol groups compared with Group C *p* = 0.020. At 120 min (R120), no statistically significant differences were observed among groups (*p* = 0.090), with all dogs approaching an alert or mildly sedated state (median 0–−1).

Inter-observer agreement for RASS scoring was high, with a Kendall’s coefficient of concordance W = 0.89 (*p* < 0.001), indicating high reproducibility and reliability of sedation assessments performed independently by the three blinded observers.

Time to extubation was not formally recorded; however, extubation was performed upon recovery of protective airway reflexes, and no clinically relevant differences were observed among groups.

Postoperative sedation gradually resolved within 2 h after extubation (Table 4).

### 3.6. Plasma Serotonin Concentrations (5-HT)

Plasma serotonin (5-hydroxytryptamine, 5-HT) at baseline, mean 5-HT levels were similar among groups with no significant differences (*p* = 0.8214) for both comparisons, confirming comparable preoperative serotonin concentrations.

At 12 h postoperatively, plasma serotonin decreased in the tapentadol-containing groups, with Group TM showing a 16.3% reduction (*p* = 0.0101) vs. C and Group T showing a 30.0% reduction (*p* < 0.0001) vs. C relative to the control group.

This trend persisted at 24 h, with Group TM and Group T maintaining lower 5-HT levels compared with Group C (*p* < 0.0001).

These results indicate that preoperative tapentadol administration, alone or in combination with meloxicam, was associated with a significant postoperative reduction in plasma serotonin concentrations, suggesting a modulatory effect of the analgesic protocols on serotonergic activity during the early postoperative period (Table 5).

Data are presented as mean ± standard deviation (SD) of plasma serotonin (5-hydroxytryptamine, 5-HT) concentrations expressed in ng/mL. Measurements were obtained in three groups: Group C (control, n = 22), Group T (n = 22), and Group TM (n = 22). Blood samples were collected at baseline (preoperative) and at 12 h and 24 h postoperatively. Diff % TM vs. C indicates the percentage difference in serotonin levels between Group TM and the control group (C) at the same time point. Diff % T vs. C indicates the percentage difference between Group T and the control group. p TM vs. C and p T vs. C represent the *p*-values for the statistical comparisons between Group TM vs. Group C and Group T vs. Group C, respectively, at each time point. Statistical significance was considered at *p* < 0.05. Values highlighted by lower *p*-values indicate statistically significant differences in plasma serotonin concentrations compared with the control group during the postoperative period.

### 3.7. Serum Biochemical Parameters

Serum biochemical parameters, including glucose, albumin, total protein, and blood urea concentrations, were evaluated at baseline (T0), 12 h, and 24 h postoperatively to assess potential metabolic or renal effects associated with anesthesia and perioperative analgesic protocols.

At baseline, all measured biochemical parameters were comparable among the three experimental groups Group C (Meloxicam + Atropine), Group TM (Tapentadol + Meloxicam + Atropine), and Group T (Tapentadol + Atropine) with no statistically significant differences observed (*p* > 0.05 for all variables). These findings confirmed homogeneity of the study population prior to the administration of the preanesthetic treatments.

A transient postoperative increase in serum glucose concentration was observed at 12 h in all groups, reflecting the physiological metabolic response to surgical stress and anesthesia. However, dogs receiving tapentadol-containing protocols (Groups TM and T) showed significantly lower glucose concentrations compared with the control group (Group C) at the same time point (*p* = 0.018). By 24 h postoperatively, serum glucose levels had decreased toward baseline values in all groups, and no significant intergroup differences were detected.

Serum albumin and total protein concentrations showed mild reductions at 12 h postoperatively in all groups, likely reflecting perioperative fluid therapy and physiological redistribution of plasma proteins. These changes remained within normal reference ranges and did not differ significantly among groups at any time point (*p* > 0.05).

Similarly, blood urea concentrations exhibited a slight transient increase at 12 h following surgery, which may be attributed to perioperative metabolic changes and mild postoperative catabolism. However, values remained within physiological limits and did not differ significantly among treatment groups during the study period.

Overall, these findings indicate that the different preanesthetic analgesic protocols did not induce clinically relevant alterations in the evaluated biochemical parameters. The results suggest that preoperative administration of tapentadol, alone or in combination with meloxicam, was well tolerated and did not adversely affect metabolic or renal indicators during the early postoperative period (Table 6).

### 3.8. Propofol and Sevoflurane Requirements

The mean propofol requirement for induction in the control group was 5.0 ± 0.3 mg/kg. Preoperative tapentadol administration resulted in a slight reduction in propofol needs, with the T group requiring 4.7 ± 0.2 mg/kg and the TM group 4.5 ± 0.3 mg/kg. Sevoflurane requirements, expressed as end-tidal concentration (CSE), were similar between the control and T groups (C: 4.0 ± 0.4%; T: 4.0 ± 0.5%), whereas the TM group exhibited a significant decrease (3.0 ± 0.8%, *p* < 0.05 vs. control). These results indicate that preoperative tapentadol, particularly in combination with multimodal analgesia (TM group), reduced intraoperative anesthetic consumption and promoted greater anesthetic stability.

## 4. Discussion

The present study evaluated the effects of preoperative tapentadol administration on anesthetic depth, postoperative recovery profile, and serotonergic modulation in dogs undergoing elective ovariectomy under propofol–sevoflurane anesthesia. The results indicate that tapentadol administration before anesthesia was associated with improved anesthetic depth during surgical stimulation, enhanced early postoperative sedation, and significant modulation of plasma serotonin concentrations without clinically relevant metabolic alterations.

Adequate perioperative analgesia is essential for maintaining physiological stability during anesthesia and optimizing postoperative recovery in veterinary surgical patients. Surgical trauma activates a complex neuroendocrine response characterized by the release of inflammatory mediators, activation of the hypothalamic–pituitary–adrenal axis, and modulation of central nociceptive pathways [24,25,26]. If inadequately controlled, this response may contribute to increased anesthetic requirements, central sensitization, and prolonged postoperative discomfort [27]. In the present study, all preanesthetic protocols provided adequate nociceptive control, as demonstrated by the low cumulative pain scores observed throughout the surgical procedure and the absence of rescue analgesia requirements.

Despite comparable analgesic efficacy among the protocols, dogs receiving tapentadol exhibited significantly deeper anesthetic planes during nociceptive surgical phases, including skin incision, laparotomy, and ovarian traction. These findings suggest that tapentadol contributed to improved hypnotic stability during anesthesia. Tapentadol is characterized by a dual mechanism of action involving μ-opioid receptor agonism and norepinephrine reuptake inhibition, which enhances descending inhibitory pain pathways and reduces central nociceptive transmission [18,28,29]. The attenuation of afferent nociceptive input to supraspinal structures may stabilize thalamocortical circuits responsible for maintaining anesthesia-induced unconsciousness, thereby promoting deeper and more consistent anesthetic states. The interaction between analgesia and anesthesia-induced sleep has become an important area of investigation in modern anesthesiology. General anesthesia produces neurophysiological states that share characteristics with deep non-rapid eye movement (NREM) sleep, including synchronized slow-wave cortical oscillations and reduced functional connectivity between cortical and subcortical structures. Propofol and volatile anesthetics such as sevoflurane induce high-amplitude slow delta oscillations within thalamocortical networks, producing EEG patterns resembling physiological slow-wave sleep [7,30,31].

The stability of these oscillatory patterns has been associated with improved anesthetic control and reduced risk of intraoperative arousal [32]. Recent experimental studies have suggested that pharmacological modulation of nociceptive input may influence the quality of anesthesia-induced sleep. In swine undergoing hernia repair, the addition of tramadol to a romifidine/ketamine–diazepam anesthetic protocol improved the depth and stability of anesthesia-induced sleep and was associated with alterations in systemic serotonin concentrations [33]. These findings support the hypothesis that monoaminergic neurotransmission plays a key role in regulating neural oscillatory activity during anesthetic-induced unconsciousness.

The postoperative sedation patterns observed in the present study further support the central neurophysiological effects of tapentadol. Dogs receiving tapentadol exhibited significantly deeper sedation during the early recovery phase following extubation compared with control animals. Although recovery dysphoria was not formally assessed, the increased early postoperative sedation observed in the tapentadol treated groups may have contributed to a smoother recovery and reduced emergence agitation. This effect was transient and gradually resolved within two hours after anesthesia. Controlled postoperative sedation may represent a beneficial phenomenon, as it reduces agitation and dysphoria commonly observed during recovery from volatile anesthetics [1,34]. Emergence agitation following inhalational anesthesia has been associated with rapid changes in cortical activity and incomplete resolution of nociceptive stimulation, both of which may be mitigated by adequate perioperative analgesia [35].

One of the most relevant findings of the present study was the modulation of plasma serotonin concentrations in dogs receiving tapentadol. While baseline serotonin levels were comparable across groups, postoperative concentrations were significantly reduced in the tapentadol-treated groups at both 12 and 24 h. Serotonin (5-hydroxytryptamine, 5-HT) plays a central role in the regulation of sleep–wake cycles, nociceptive processing, autonomic function, and neuroendocrine homeostasis [13,36]. Serotonergic neurons located in the raphe nuclei project extensively throughout the central nervous system and interact with thalamocortical circuits responsible for regulating arousal and sleep synchronization [37].

Experimental evidence suggests that serotonergic signaling contributes to the modulation of cortical oscillatory activity during both natural sleep and anesthesia-induced unconsciousness [38]. Alterations in serotonin levels may therefore reflect neurochemical adaptations associated with surgical stress and anesthetic exposure. In dogs undergoing ovariectomy under general anesthesia, systemic serotonin fluctuations have been correlated with oxidative stress markers and inflammatory responses, suggesting that serotonergic pathways participate in the neurochemical stress response associated with surgery [39].

The reduction in serotonin concentrations observed in the tapentadol-treated dogs in the present study may therefore reflect attenuation of surgical stress responses through improved analgesic control. Similar neurochemical patterns have been described in horses with chronic joint disorders treated with tapentadol, where decreased plasma serotonin concentrations were associated with reduced adrenocortical activity and improved pain control [40]. These findings support the hypothesis that tapentadol may modulate neuroendocrine pathways involved in stress and nociception beyond its direct analgesic effects.

Monoaminergic systems, including serotonergic and noradrenergic pathways, play a crucial role in descending pain modulation and autonomic regulation. Activation of these pathways can suppress nociceptive transmission at the spinal level while simultaneously influencing central arousal networks and emotional responses to pain [41]. The norepinephrine reuptake inhibition component of tapentadol may therefore contribute not only to analgesic efficacy but also to stabilization of central autonomic and neurochemical responses during anesthesia and recovery.

The biochemical analyses performed in this study further confirmed the safety of the evaluated analgesic protocols. Although a transient increase in serum glucose was observed postoperatively in all groups, this response is consistent with the physiological metabolic reaction to surgical stress and anesthesia [42]. Interestingly, dogs receiving tapentadol exhibited significantly lower glucose concentrations at 12 h compared with control animals, suggesting partial attenuation of the metabolic stress response. Effective perioperative analgesia has been shown to reduce activation of the hypothalamic–pituitary–adrenal axis and limit stress-induced hyperglycemia [43]. In Group T, effective analgesia was maintained for up to 6 h postoperatively without additional NSAID administration, suggesting that preoperative tapentadol alone provided sufficient early postoperative analgesic coverage.

No clinically relevant differences were observed for albumin, total protein, or blood urea concentrations among the groups. Mild transient reductions in plasma protein concentrations are commonly observed following surgical procedures due to perioperative fluid therapy and physiological redistribution of plasma proteins [44]. The absence of significant alterations in renal or metabolic parameters indicates that tapentadol administration was well tolerated and did not induce systemic adverse effects during the early postoperative period.

Collectively, the results of this study suggest that preoperative tapentadol administration may enhance the neurophysiological quality of anesthesia-induced sleep while simultaneously modulating neurochemical pathways associated with stress and nociceptive processing. The integration of serotonergic biomarkers with clinical anesthetic parameters represents a promising approach for improving the understanding of perioperative neurophysiology in veterinary medicine. Nevertheless, several limitations should be acknowledged. First, anesthetic depth was evaluated using clinical parameters rather than electroencephalographic monitoring, which would provide more detailed characterization of neural oscillatory dynamics during anesthesia. Second, plasma serotonin concentrations may not directly reflect central serotonergic activity within the brain. Pupil size may have been influenced by atropine administration, potentially limiting its reliability as an indicator of anesthetic depth. Future investigations combining EEG-based anesthetic monitoring with neurochemical and endocrine biomarkers could provide deeper insights into the mechanisms linking analgesia, anesthesia-induced sleep, and perioperative neurochemical homeostasis.

## 5. Conclusions

Preoperative administration of tapentadol, at the dose used in this study, in dogs undergoing elective ovariectomy under propofol–sevoflurane anesthesia resulted in a deeper and more stable anesthetic plane during nociceptive surgical stimulation and produced a transient increase in early postoperative sedation without impairing recovery quality. Tapentadol-treated dogs also exhibited significant postoperative modulation of plasma serotonin concentrations, suggesting an interaction between analgesic mechanisms and serotonergic pathways involved in sleep regulation, nociceptive processing, and neuroendocrine stress responses. The analgesic protocols evaluated maintained effective intraoperative and postoperative pain control and did not induce clinically relevant metabolic or biochemical alterations. Overall, these findings support the integration of tapentadol within multimodal perioperative analgesic strategies in canine anesthesia and highlight the potential role of serotonergic modulation as a biomarker of anesthetic stability and postoperative recovery.

## Figures and Tables

**Table 1:** Intraoperative cumulative pain scores (CPSs) in dogs receiving different preanesthetic protocols during ovariectomy under propofol–sevoflurane anesthesia. Values are expressed as median (range).

Surgical Time Point	Group C (Meloxicam + Atropine)	Group TM (Tapentadol + Meloxicam + Atropine)	Group T (Tapentadol + Atropine)	*p*-Value
**Post-induction (A)**	0 (0–2)	0 (0–1)	0 (0–1)	0.45
**Skin incision (SI)**	2 (1–3)	1 (0–2)	1 (0–2)	0.12
**Laparotomy (L)**	2 (1–3)	1 (0–2)	1 (0–2)	0.10
**Ovarian traction (TPI–TPII)**	3 (2–4)	2 (1–3)	2 (1–3)	0.08
**Skin closure (SC)**	1 (0–2)	0 (0–1)	0 (0–1)	0.15
**Median total CPS**	2 (0–4)	1 (0–3)	1 (0–3)	–

CPS = cumulative pain score, calculated as the sum of percentage changes in HR, RR, and systolic arterial pressure relative to post-induction values (0–4 per variable; max 12 per time point). No dogs required rescue analgesia (fentanyl 2 µg kg^−1^ IV) during the procedure. Kruskal–Wallis test, followed by Dunn’s post hoc test; *p* < 0.05 considered significant. Analysis revealed a high degree of concordance between observers, with W = 0.91 (*p* < 0.001), indicating excellent inter-observer reliability.

**Table 2:** Total anesthetic depth scores at predefined intraoperative time points in dogs undergoing ovariectomy under propofol–sevoflurane anesthesia.

Time Point	Group C (Meloxicam + Atropine)	Group TM (Tapentadol + Meloxicam + Atropine)	Group T (Tapentadol + Atropine)	*p*-Value
**Post-induction (A)**	7 (6–8)	8 (7–9)	8 (7–9)	0.12
**Skin incision (SI)**	5 (4–6)	7 (6–8) *	7 (6–8) *	0.003
**Laparotomy (L)**	5 (4–6)	7 (6–8) *	7 (6–8) *	0.002
**Ovarian traction (TPI–TPII)**	4 (3–5)	6 (5–7) *	6 (5–7) *	0.001
**Skin closure (SC)**	6 (5–7)	7 (6–8)	7 (6–8)	0.08
**Median total**	5 (3–8)	7 (5–9)	7 (5–9)	-
**Range total**	3–8	5–9	5–9	-

Scores are presented as median (range) for each experimental group: Group C (Meloxicam + Atropine), Group TM (Tapentadol + Meloxicam + Atropine), and Group T (Tapentadol + Atropine). Anesthetic depth was evaluated using a composite scoring system based on palpebral reflex, corneal reflex, jaw tone, eye position, and pupil size at post-induction (A), skin incision (SI), laparotomy (L), traction of the first and second ovary (TPI, TPII), and skin closure (SC). Significant differences between groups at specific time points are indicated (* *p* < 0.05, Kruskal–Wallis test with Dunn’s post hoc test). Overall, dogs in the tapentadol-containing groups (TM and T) exhibited deeper planes of anesthesia during nociceptive stimuli (SI, L, TPI, TPII) compared with Group C. Inter-observer agreement, for anesthetic depth scoring, was high, with Kendall’s coefficient of concordance W = 0.92 (*p* < 0.001), confirming the reliability and reproducibility of the clinical assessments performed by blinded observers.

**Table 3:** Postoperative pain scores (median, range) in dogs receiving different preanesthetic protocols during elective ovariectomy. Values are presented at extubation and at 6-h intervals up to 24 h.

Time Point	Group C (Meloxicam + Atropine)	Group TM (Tapentadol + Meloxicam + Atropine)	Group T (Tapentadol + Atropine)
**Extubation (R0)**	1 (0–2)	1 (0–2)	1 (0–2)
**6 h**	1 (0–2)	1 (0–2)	1 (0–2)
**12 h**	1 (0–1)	1 (0–1)	1 (0–1)
**18 h**	0 (0–1)	0 (0–1)	0 (0–1)
**24 h**	0 (0–1)	0 (0–1)	0 (0–1)

**Table 4:** Postoperative sedation scores assessed using a modified Richmond Agitation–Sedation Scale (RASS) in dogs undergoing ovariectomy under propofol–sevoflurane anesthesia following different preanesthetic analgesic protocols. Values are presented as median (range).

Time Point	Group C (Meloxicam + Atropine)	Group TM (Tapentadol + Meloxicam + Atropine)	Group T (Tapentadol + Atropine)	*p*-Value
R0 (extubation)	−1 (−2 to 0)	−3 (−4 to −2) *	−3 (−4 to −2) *	0.004
R30	−1 (−2 to 0)	−2 (−3 to −1) *	−2 (−3 to −1) *	0.006
R60	0 (−1 to 0)	−1 (−2 to 0) *	−1 (−2 to 0) *	0.020
R120	0 (0 to 1)	−1 (−1 to 0)	−1 (−1 to 0)	0.090

R0 = immediately after extubation; R30 = 30 min after extubation; R60 = 60 min after extubation; R120 = 120 min after extubation. Sedation was assessed using a modified Richmond Agitation–Sedation Scale (RASS), where scores range from +4 (combative) to −5 (unarousable), with negative values indicating increasing depth of sedation. Data were analyzed using the Kruskal–Wallis test followed by Dunn’s multiple comparison post hoc test. * Significantly different from Group C (*p* < 0.05).

**Table 5:** Plasma serotonin concentrations (5-HT, ng/mL; mean ± SD) in the three experimental groups at different postoperative time points.

Time	Group C (n = 22)	Group T (n = 22)	Group TM (n = 22)	Diff % TM vs. C	Diff % T vs. C	p TM vs. C	p TT vs. C
**Baseline**	20.90 ± 2.00	21.10 ± 2.10	20.70 ± 2.00	+1.0%	−1.0%	0.8214	0.8214
**12 h**	27.01 ± 4.00	22.60 ± 3.20	18.90 ± 2.50	−16.3%	−30.0%	0.0101	<0.0001
**24 h**	26.80 ± 3.50	21.90 ± 3.00	17.60 ± 2.80	−18.3%	−34.3%	0.0022	<0.0001

**Table 6:** Serum Biochemical Parameters.

Parameter	Time	Group C (Meloxicam + Atropine)	Group TM (Tapentadol + Meloxicam + Atropine)	Group T (Tapentadol + Atropine)	*p*-Value
**Serum glucose (mg/dL)**	T0	94.8 ± 8.2	95.1 ± 8.5	94.6 ± 8.4	0.93
	12 h	108.6 ± 10.4	101.2 ± 9.6*	102.8 ± 9.9 *	0.018
	24 h	100.3 ± 9.1	97.4 ± 8.8	**98**.1 ± 9.0	0.29
**Albumin (g/dL)**	T0	3.43 ± 0.29	3.40 ± 0.27	3.41 ± 0.28	0.88
	12 h	3.29 ± 0.26	3.33 ± 0.25	3.35 ± 0.24	0.54
	24 h	3.31 ± 0.27	3.36 ± 0.26	3.37 ± 0.25	0.49
**Total protein (g/dL)**	T0	6.38 ± 0.44	6.34 ± 0.46	6.36 ± 0.45	0.91
	12 h	6.10 ± 0.41	6.18 ± 0.40	6.20 ± 0.42	0.46
	24 h	6.18 ± 0.39	6.25 ± 0.41	6.26 ± 0.40	0.52
**Blood urea (mg/dL)**	T0	31.2 ± 4.6	30.9 ± 4.8	31.0 ± 4.7	0.95
	12 h	34.6 ± 5.2	32.8 ± 5.0	33.1 ± 5.1	0.34
	24 h	32.9 ± 4.9	31.7 ± 4.7	31.9 ± 4.8	0.57

T0 = baseline before premedication; Group C = Meloxicam + Atropine; Group TM = Tapentadol + Meloxicam + Atropine; Group T = Tapentadol + Atropine; * Significantly different vs. Group C at the same time point (*p* < 0.05).

## Data Availability

The original contributions presented in this study are included in the article. Further inquiries can be directed to the corresponding authors.
